# *In Vitro* Activity Comparison of Ceftazidime–Avibactam and Aztreonam–Avibactam Against Bloodstream Infections With Carbapenem-Resistant Organisms in China

**DOI:** 10.3389/fcimb.2021.780365

**Published:** 2021-11-25

**Authors:** Wei Yu, Luying Xiong, Qixia Luo, Yunbo Chen, Jinru Ji, Chaoqun Ying, Zhiying Liu, Yonghong Xiao

**Affiliations:** State Key Laboratory for Diagnosis and Treatment of Infectious Diseases, National Clinical Research Center for Infectious Diseases, Collaborative Innovation Center for Diagnosis and Treatment of Infectious Diseases, The First Affiliated Hospital, Zhejiang University School of Medicine, Hangzhou, China

**Keywords:** carbapenem-resistant Enterobacterales (CRE), *Pseudomonas aeruginosa*, carbapenemase, avibactam, bloodstream infections

## Abstract

**Objectives:**

The aim of this work was to investigate the activity of ceftazidime–avibactam (CZA) and aztreonam–avibactam (AZA) against bloodstream infections caused by carbapenem-resistant organisms (CROs).

**Methods:**

Non-duplicate CROs, including 56 carbapenem-resistant *Escherichia coli* (CR-Eco), 318 carbapenem-resistant *Klebsiella pneumoniae* (CR-Kpn), and 65 carbapenem-resistant *Pseudomonas aeruginosa* (CR-Pae), were collected using the Blood Bacterial Resistant Investigation Collaborative System (BRICS) program in China. The minimum inhibitory concentrations (MICs) of 24 antibiotics were tested. Carbapenemase genes were amplified for CZA-resistant CROs by PCR. The MICs of CZA and AZA were further determined with avibactam at 8 and 16 mg/L, respectively.

**Results:**

The resistance rate of polymyxin B against CROs was less than 5%. Only one CR-Kpn was resistant to tigecycline. The resistance rates of CZA against CR-Eco, CR-Kpn, and CR-Pae were 75.0%, 12.6%, and 18.5%, respectively. The MIC_90_ values of AZA against CR-Eco, CR-Kpn, and CR-Pae were 2/4, 1/4, and 64/4 mg/L, respectively. Among the CZA-resistant CROs, 42 (100%) CR-Eco, 24 (60%) CR-Kpn, and 1 (8.3%) CR-Pae isolates harbored metallo-β-lactamase genes. The increase of avibactam concentration enhanced the susceptibility of CZA and AZA against CROs, especially for CR-Eco and CR-Kpn.

**Conclusions:**

The *in vitro* activity of AZA was superior to that of CZA against CR-Eco and CR-Kpn, whereas CZA showed better effect against CR-Pae.

## Introduction

Carbapenem-resistant organisms (CROs) have become a global epidemic problem for many years. The reported rate of carbapenem resistance in non-fermenters, such as *Pseudomonas aeruginosa* and *Acinetobacter baumanii*, was higher than that in Enterobacterales (Chamieh et al., 2020). It is of note that the carbapenem resistance rates among the different bacterial isolation sites showed differences, such as the rates for carbapenem-resistant *P. aeruginosa* (CR-Pae) and carbapenem-resistant *A. baumannii* in bloodstream infections (BSIs) that were lower than those in respiratory infections (Cai et al., 2017). In China, the proportions of *Escherichia coli* (9.8%–13.6%) and *Klebsiella pneumoniae* (5.3%–10.4%) in BSIs increased significantly from 2010 to 2019, while the proportion of *P. aeruginosa* decreased significantly from 4.0% to 2.4% (Cui et al., 2021). Current evidence revealed that carbapenemase and β-lactamases combined with mutations that decrease permeability were associated with carbapenem resistance (Queenan and Bush, 2007).

Patients with bloodstream infections caused by carbapenem-resistant organisms (BSIs-CROs) suffer from a high risk of mortality, emphasizing the need for novel and rational therapies (Lemos et al., 2014; Martin et al., 2018). Several novel β-lactam/β-lactamase inhibitor combinations have been developed against various CROs, such as ceftazidime–avibactam (CZA), aztreonam–avibactam (AZA), meropenem–vaborbactam, and imipenem/cilastatin–relebactam (Papp-Wallace, 2019). Avibactam, as a bridged diazabicyclo[3.2.1]octanone (DBO) non-β-lactam inhibitor, provides excellent inhibition of class A, class C, and some of the class D β-lactamases (Bush and Bradford, 2019). Recently, CZA represented an important advance in the treatment of infections caused by CR-Pae and carbapenem-resistant Enterobacteriaceae (CRE) (Onorato et al., 2019). However, the activity of CZA against metallo-β-lactamases (MBLs) was limited (Bush and Bradford, 2019). Notably, AZA has been shown to be a potential treatment to inhibit MBLs (Biagi et al., 2019). Therefore, this study aimed to compare the *in vitro* activity of these two avibactam combinations (CZA and AZA) against BSIs-CROs.

## Materials and Methods

### Bacterial Isolates

Carbapenem resistance is defined as isolates resistant to imipenem, meropenem, or ertapenem, according to the Clinical and Laboratory Standards Institute (CLSI) interpretation (Clinical and Laboratory Standards Institute, 2020). A total of non-duplicate 56 carbapenem-resistant *E. coli* (CR-Eco), 318 carbapenem-resistant *K. pneumoniae* (CR-Kpn), and 65 CR-Pae were collected using the Blood Bacterial Resistant Investigation Collaborative System (BRICS) program in 2019 from 40 hospitals in China.

### Antimicrobial Susceptibility Testing

The minimum inhibitory concentrations (MICs) of 24 antibiotics [cefazolin, cefuroxime, ceftriaxone, ceftazidime (CAZ), cefepime, cefoxitin, moxalactam, aztreonam (ATM), ertapenem, imipenem, meropenem, amoxicillin–clavulanic acid, piperacillin–tazobactam, cefoperazone–sulbactam, CZA, AZA, gentamicin, amikacin, ciprofloxacin, levofloxacin, fosfomycin, tigecycline, polymyxin B, and trimethoprim–sulfamethoxazol] were tested for CR-Eco and CR-Kpn. In addition, 14 antibiotics (CAZ, cefepime, ATM, imipenem, meropenem, piperacillin–tazobactam, cefoperazone–sulbactam, CZA, AZA, gentamicin, amikacin, ciprofloxacin, levofloxacin, and polymyxin B) were measured for CR-Pae. Polymyxin B and glucose-6-phosphate were obtained from Sigma-Aldrich (St. Louis, MO, USA); the other antibiotics were purchased from Dalian Meilun Biotech (Dalian, China). Broth microdilution was used for tigecycline and polymyxin B, while the agar dilution method was used for the other 22 antibiotics according to CLSI (Clinical and Laboratory Standards Institute, 2012; Clinical and Laboratory Standards Institute, 2020). *E. coli* ATCC 25922, *K. pneumoniae* ATCC BAA-1705, and *P. aeruginosa* ATCC 27853 were used as quality control.

The MIC_50_ and MIC_90_ (the MIC required to inhibit the growth of 50% and 90%, respectively, of the population) values were calculated for the 24 antibiotics. The MIC distribution of CAZ, CZA, ATM, and AZA was represented by cumulative inhibition ratio (CIR) curves.

### Carbapenemase Genes of CZA-Resistant CROs

The definition of CZA resistance was referred to the CLSI (Clinical and Laboratory Standards Institute, 2020). Carbapenemase genes (*bla*_IMP_, *bla*_SPM_, *bla*_AIM_, *bla*_VIM_, *bla*_GIM_, *bla*_SIM_, *bla*_NDM_, *bla*_DIM_, and *bla*_KPC_) were amplified by PCR and sequenced with Sanger dideoxy-mediated chain termination for CZA-resistant CROs (Poirel et al., 2011). Each PCR was completed in triplicate.

### MICs of CAZ and ATM With Increased Avibactam Concentration Against CZA-Resistant CROs and CR-Pae With High-Level MIC of AZA

The MICs of CAZ and ATM combined with avibactam at 8 and 16 mg/L were further tested against CZA-resistant CROs and CR-Pae with a high-level inhibitory concentration of AZA (MIC ≥ 32 mg/L).

## Results

### Geographical Distribution of BSIs-CROs

**Figure 1:**
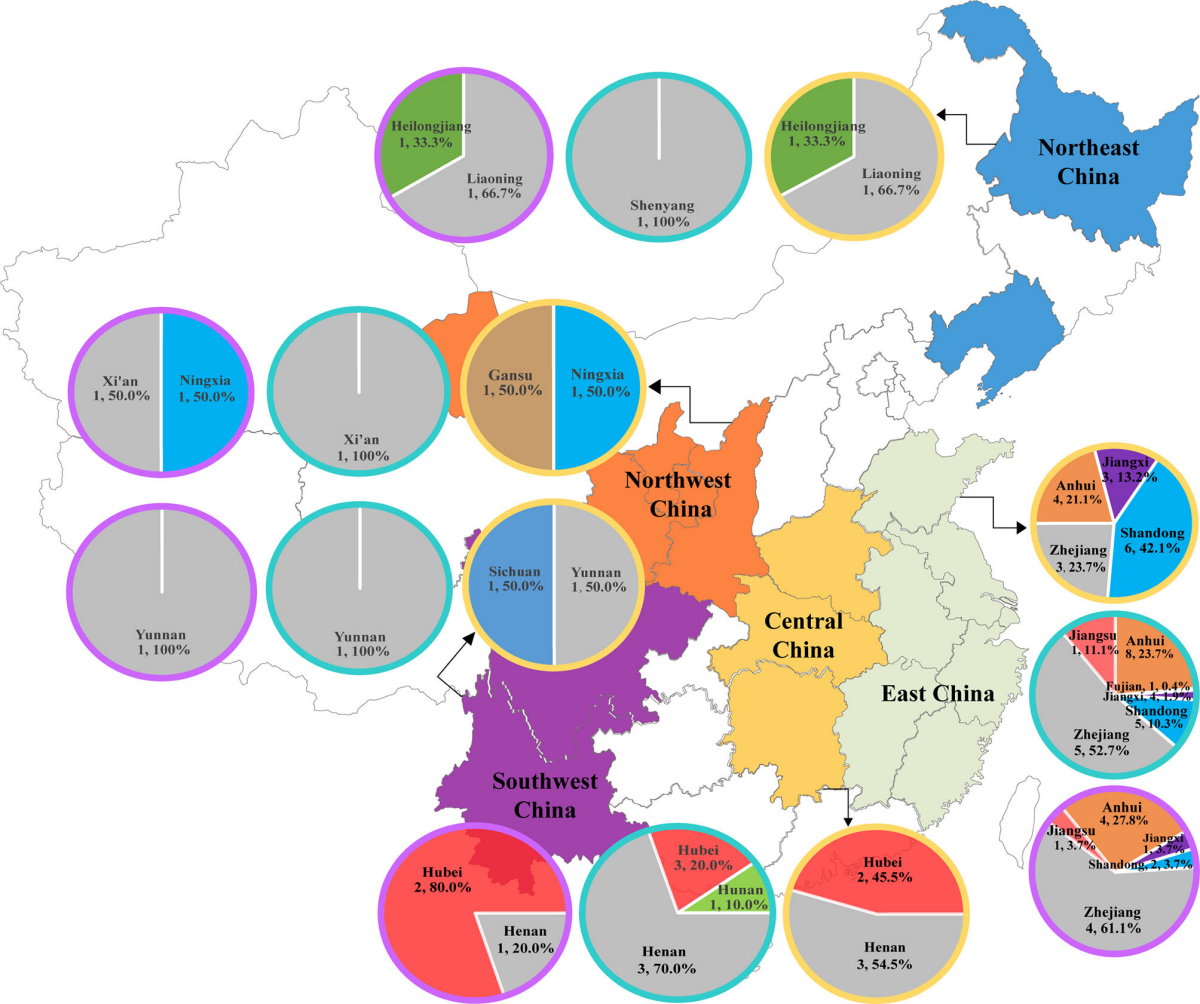
Distribution of bloodstream infections (BSIs) due to carbapenem-resistant organisms (CROs) in China. The description included the regions, number of hospitals, and the proportions of isolates in the corresponding regions. *CR-Eco*, carbapenem-resistant *Escherichia coli*; *CR-Kpn*, carbapenem-resistant *Klebsiella pneumoniae*; *CR-Pae*, carbapenem-resistant *Pseudomonas aeruginosa*. *Yellow circle*, CR-Eco; *blue circle*, CR-Kpn; *purple circle*, CR-Pae.

CR-Eco, CR-Kpn, and CR-Pae isolates were collected from 27, 34, and 20 hospitals, respectively (**Figure 1**). Most strains were isolated from East China (EC) and Central China (CC) due to the majority of the involved hospitals located in these areas. A total of 38 CR-Eco, 262 CR-Kpn, and 54 CR-Pae isolates were from EC. In addition, there were 11 CR-Eco, 30 CR-Kpn, and 5 CR-Pae isolates collected from CC.

### Antibiotic Susceptibility Test

A summary of the MICs is shown in **Table 1**. All CR-Eco isolates were susceptible to tigecycline. One CR-Eco and 13 CR-Kpn isolates were resistant to polymyxin B. There were 97.2% BSIs CR-Kpn isolates susceptible to tigecycline. Resistance to amikacin was observed in one CR-Pae isolate. In addition, all CR-Pae isolates were intermediate to polymyxin B due to no susceptible breakpoint for polymyxin B in the CLSI criterion. It is of note that 44.6% and 36.9% of the CR-Pae isolates remained susceptible to CAZ and ATM, respectively. However, the susceptibility rates of CAZ and ATM were lower in CRE, especially for CR-Kpn. The addition of avibactam to CAZ and ATM restored the activity against CROs (**Supplementary Figure S1**). The resistance rates of CZA against CR-Eco, CR-Kpn, and CR-Pae were 75.0%, 12.6%, and 18.5%, respectively. Currently, the susceptibility breakpoint for AZA has not been approved. The MIC_90_ values of AZA against CR-Eco, CR-Kpn, and CR-Pae were 2/4, 1/4, and 64/4 mg/L, respectively.

**Table 1:** Antibiotic susceptibility test of the 24 antibiotics.

Antibiotics	CR-Eco					CR-Kpn					CR-Pae				
	MIC range (mg/L)	MIC_50_ (mg/L)	MIC_90_ (mg/L)	S	R	MIC range (mg/L)	MIC_50_ (mg/L)	MIC_90_ (mg/L)	S	R	MIC range (mg/L)	MIC_50_ (mg/L)	MIC_90_ (mg/L)	S	R
				*N*, (%)	*N*, (%)				*N*, (%)	*N*, (%)				*N*, (%)	*N*, (%)
Cefazolin	128	128	128	0 (0.0)	56 (100.0)	1–128	128	128	2 (0.6)	315 (99.1)	–	–	–	–	–
Cefuroxime	32–128	128	128	0 (0.0)	56 (100.0)	1–128	128	128	3 (0.9)	314 (98.7)	–	–	–	–	–
Ceftriaxone	16–128	64	64	0 (0.0)	56 (100.0)	0.125–128	64	64	2 (0.6)	316 (99.4)	–	–	–	–	–
Ceftazidime	16–128	64	64	0 (0.0)	56 (100.0)	1–64	64	64	1 (0.3)	313 (98.4)	2–64	16	64	29 (44.6)	29 (44.6)
Cefepime	0.06–64	64	64	1 (1.8)	53 (94.6)	0.03–128	64	64	5 (1.6)	294 (92.4)	1–64	8	64	35 (53.8)	25 (38.5)
Cefoxitin	8–128	128	128	1 (1.8)	55 (98.2)	1–128	128	128	4 (1.3)	309 (97.2)	–	–	–	–	–
Moxalactam	2–128	128	128	1 (1.8)	53 (94.6)	0.25–128	128	128	16 (5.0)	288 (90.6)	–	–	–	–	–
Aztreonam	0.125–128	64	64	10 (17.9)	41 (73.2)	0.125–64	64	64	16 (5.0)	301 (94.6)	2–64	32	64	24 (36.9)	35 (53.8)
Ertapenem	4–32	64	64	0 (0.0)	56 (100.0)	2–32	32	32	0 (0)	318 (100.0)	–	–	–	–	–
Imipenem	1–32	8	32	1 (1.8)	54 (96.4)	0.5–32	32	32	1 (0.3)	316 (99.4)	0.5–32	32	32	6 (9.2)	57 (87.7)
Meropenem	0.5–32	8	32	1 (1.8)	53 (94.6)	0.5–32	32	32	1 (0.3)	314 (98.7)	8–32	32	32	0 (0.0)	65 (100.0)
AMC (2:1)	8/4–128/64	128/64	128/64	1 (1.8)	54 (96.4)	16/8–128/64	128/64	128/64	0 (0)	316 (99.4)	–	–	–	–	–
TZP	2/4–128/4	128/4	128/4	5 (8.9)	42 (75.0)	4/4–256/4	128/4	128/4	2 (0.6)	293 (92.1)	2/4–128/4	64/4	128/4	31 (47.7)	31 (47.7)
CSL (2:1)	1/0.5–128/64	128/64	128/64	2 (3.6)	52 (92.9)	0.5/0.25–128/64	128/64	128/64	1 (0.3)	314 (98.7)	4/2–128/64	64/32	128/64	24 (36.9)	33 (50.8)
CZA	0.06/4 to >64/4	>64/4	>64/4	14 (25.0)	42 (75.0)	0.5/4 to >64/4	4/4	128/4	278 (87.4)	40 (12.6)	1/4 to >64/4	4/4	16/4	53 (81.5)	12 (18.5)
AZA	<0.015/4 to >128/4	0.5/4	2/4	NA	NA	<0.015/4 to >128/4	0.5/4	1/4	NA	NA	0.25/4–128/4	16/4	64/4	NA	NA
Gentamicin	0.25–128	64	128	18 (32.1)	36 (64.3)	0.25–128	128	128	63 (19.8)	251 (78.9)	0.5–128	4	8	46 (70.8)	4 (6.2)
Amikacin	0.5–128	4	128	46 (82.1)	9 (16.1)	0.25–128	128	128	107 (33.6)	209 (65.7)	1–128	2	4	64 (98.5)	1 (1.5)
Ciprofloxacin	0.03–32	32	32	2 (3.6)	54 (96.4)	0.007–32	32	32	9 (2.8)	308 (96.9)	0.125–32	8	32	20 (30.8)	43 (66.2)
Levofloxacin	0.125–32	32	32	2 (3.6)	52 (92.9)	0.125–32	32	32	11 (3.5)	298 (93.7)	0.5–128	8	32	21 (32.3)	37 (56.9)
Fosfomycin	0.5–256	1	128	42 (75.0)	8 (14.3)	0.5–256	32	256	203 (63.8)	97 (30.5)	–	–	–	–	–
Tigecycline	0.125–1	0.25	0.25	56 (100.0)	0 (0.0)	0.125–8	0.25	1	309 (97.2)	1 (0.3)	–	–	–	–	–
Polymyxin B	0.25–32	0.5	1	55 (98.2)	1 (1.8)	0.25–32	0.5	1	303 (95.3)^a^	15 (4.7)	0.5–2	1	2	65 (100.0)^a^	0 (0)
SXT	0.125/2.375–8/512	8/512	8/512	8 (14.3)	48 (85.7)	0.125/2.375–8/152	8/152	8/152	98 (30.8)	220 (69.2)	–				

S, susceptible; R, resistant; CZA, ceftazidime–avibactam; AZA, aztreonam–avibactam; AMC, amoxicillin–clavulanic acid; TZP, piperacillin–tazobactam; CSL, cefoperazone–sulbactam; SXT, trimethoprim–sulfamethoxazol; MIC, minimum inhibitory concentration; CR-Eco, carbapenem-resistant Escherichia coli; CR-Kpn, carbapenem-resistant Klebsiella pneumoniae; CR-Pae, carbapenem-resistant Pseudomonas aeruginosa; NA, not available.

a Intermediary to polymyxin B.

### Carbapenemase Genotype of CZA-Resistant CROs

Screening of the CZA-resistant CR-Eco isolates (42, 75%) revealed that three isolates coexisted with two carbapenemase genes (*bla*_IMP_ and *bla*_NDM_), whereas the other 39 isolates harbored *bla*_NDM_.

Among the CZA-resistant CR-Kpn isolates (40, 12.6%), 3 (7.5%), 14 (35%), and 16 (40%) isolates were positive for *bla*_IMP_, *bla*_KPC_, and *bla*_NDM_, respectively. Five isolates (12.5%) were in coexistence with two carbapenemase genes. Two isolates co-harbored *bla*_IMP_ and *bla*_NDM_, and another three isolates carried *bla*_KPC_ and *bla*_NDM_. The other two isolates were not detected in any tested carbapenemase genes.

For the CZA-resistant CR-Pae (12, 18.5%), one isolate harbored *bla*_IMP_ and four isolates carried *bla*_KPC_. However, the other seven isolates were not found in the tested carbapenemase genes (**Supplementary Table S1**).

### MICs of CAZ and ATM With Increased Avibactam Concentration Against CZA-Resistant CROs and CR-Pae With High-Level MIC of AZA

**Figure 2:**
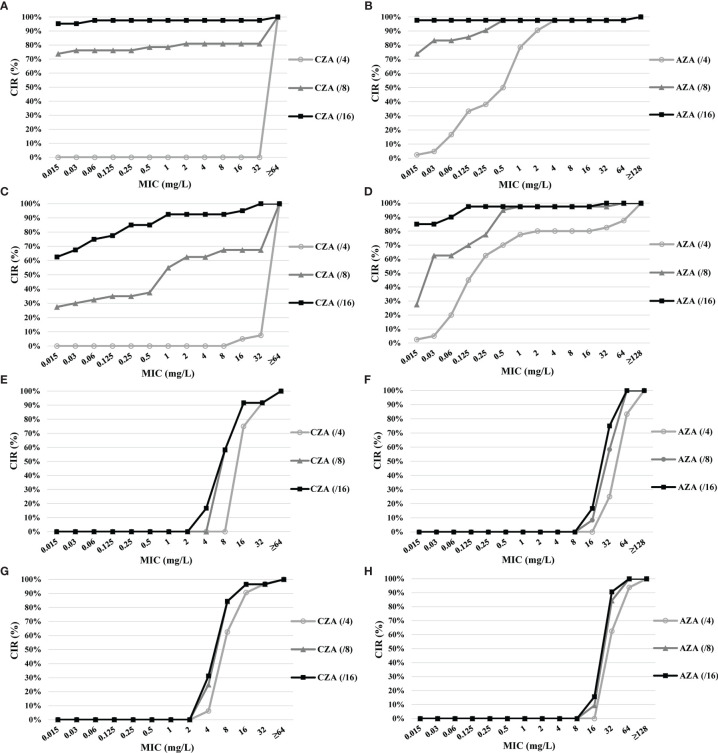
Cumulative inhibition ratios (CIRs) of ceftazidime (CAZ) and aztreonam (ATM) with increased avibactam concentration against ceftazidime–avibactam (CZA)-resistant carbapenem-resistant organisms (CROs) and carbapenem-resistant *Pseudomonas aeruginosa* (CR-Pae) with a high-level minimum inhibitory concentration (MIC) of aztreonam–avibactam (AZA). **(A)** CZA against CZA-resistant carbapenem-resistant *Escherichia coli* (CR-Eco). **(B)** AZA against CZA-resistant CR-Eco. **(C)** CZA against CZA-resistant carbapenem-resistant *Klebsiella pneumoniae* (CR-Kpn). **(D)** AZA against CZA-resistant CR-Kpn. **(E)** CZA against CZA-resistant CR-Pae. **(F)** AZA against CZA-resistant CR-Pae. **(G)** CZA against CR-Pae with the high-level MIC of AZA. **(H)** AZA against CR-Pae with the high-level MIC of AZA.

The CIRs of CZA and AZA with increased avibactam concentration are shown in **Figure 2**. Among the 42 CZA-resistant CR-Eco, the MIC of CZA above 64 mg/L was found in eight isolates with avibactam of 8 mg/L and one isolate with avibactam of 16 mg/L. The MICs of AZA against 41 CZA-resistant CR-Eco were below 0.5 mg/L with avibactam at 8 and 16 mg/L.

Of the 40 CZA-resistant CR-Kpn, 13 (32.5%) isolates with avibactam at 8 mg/L were observed resistant to CZA, while 37 (92.5%) isolates were susceptible to CZA with avibactam of 16 mg/L. The lower MICs of AZA (≤1 and ≤0.125 mg/L) accounted for 97.5% (39/40) for avibactam of 8 and 16 mg/L, respectively.

The susceptibility rate to CZA of 12 CZA-resistant CR-Pae with 8 and 16 mg/L was 58.3% (7/12). However, the MIC of AZA was higher than 32 mg/L in 11 isolates with 8 mg/L avibactam and 10 isolates with 16 mg/L avibactam.

Among the 32 CR-Pae isolates with high-level MICs of AZA, 62.5% isolates remained susceptible to CZA with avibactam at 4 mg/L. In addition, the rates of susceptibility to CZA (from 62.5% to 84.4%) and AZA (from 0% to 15.6%) increased as the avibactam concentration increased.

## Discussion

CROs have been implicated in poorer clinical outcomes than are non-CROs (Lemos et al., 2014; Martin et al., 2018). The approval of new β-lactam/β-lactamase inhibitor combinations against CROs has expanded the options for novel therapeutics (Papp-Wallace, 2019). In our study, AZA showed a much higher antibacterial activity against CRE than did CZA. However, the *in vitro* antibacterial activity of CZA against CR-Pea was superior to that of AZA. In addition, increased concentration of avibactam enhanced the susceptibility of CZA and AZA to CZA-resistant CROs, especially for CRE.

In the present study, CZA showed a higher antibacterial activity against CR-Kpn (87.4%) than against CR-Eco (25.0%) and CR-Pae (81.5%). The susceptibility rate to CZA of CRE was in keeping with the results of a previous study (Yin et al., 2019). However, the susceptibility rate of CR-Pae was higher than that found in a previous study (81.5% *vs*. 68.0%). This may be due to the different sources of isolates. Carbapenemase genes revealed that *bla*_NDM_ was common in CZA-resistant CRE, which was also consistent with other studies (Sader et al., 2017; Yin et al., 2019). In addition, 35% of the CZA-resistant CR-Kpn harbored *bla*_KPC_. Current evidence suggests that the overexpression of *bla*_KPC_ played an important role in CZA resistance (Shen et al., 2017). Interestingly, increased concentration of avibactam improved the *in vitro* activity of CZA against CRE. These results indicated that CZA with avibactam at 4 mg/L had better activity against *K. pneumoniae* carbapenemase (KPC)-producing CRE, but not against *bla*_NDM_-positive isolates, while CZA with avibactam at 8 and 16 mg/L was active against both *bla*_KPC_-positive and *bla*_NDM_-positive isolates. However, current studies have demonstrated that avibactam did not present *in vitro* activity against MBL-producing isolates (Yahav et al., 2020). There are few related studies to explain this phenomenon. Therefore, further investigations are needed to evaluate the mechanism of CZA against New Delhi metallo-β-lactamase (NDM)-producing CRE isolates.

The novel combination AZA is known to be relatively stable against both serine carbapenemases and MBL hydrolysis (Cornely et al., 2020). In our study, the MIC_90_ values of AZA against CR-Eco and CR-Kpn were 2/4 and 1/4 mg/L, respectively, which are similar to the results of a previous study (Sader et al., 2021). Likewise, a better *in vitro* antibacterial activity of AZA against CRE, especially for CR-Eco, was observed as the concentration of avibactam increased. However, the susceptibility rate of CR-Pae to AZA was lower than that to CZA in this study. Comparable susceptibility results have been reported as well (Wang et al., 2014; Karlowsky et al., 2017). Seven (58.3%) CZA-resistant CR-Pae isolates were negative for the tested carbapenemase genes. A previous study demonstrated that an upregulation of the efflux systems could result in resistance as well (Masuda et al., 2000). Thus, other mechanisms may have resulted in the high-level MIC of AZA. Fortunately, CZA was still active against 62.5% of CR-Pae with a high-level MIC of AZA. In addition, a further test confirmed that the *in vitro* antibacterial activity of CZA against CR-Pae with a high-level MIC of AZA was improved with increased concentration of avibactam. Therefore, employing the correlation of the clinical outcomes in different dosing regimens with resistance genotypes in BSIs by CR-Pae should be considered.

This study provides an insight into the activity of CZA and AZA against BSIs-CROs. However, there are also several limitations. Firstly, the isolates were only collected from China, especially in EC, which may be different from the rest of the world. Secondly, the majority of the isolates were CR-Kpn. Thirdly, the surveillance data were for 1 year, so it could not comprehensively reflect the dynamic trends of CROs.

## Conclusions

In conclusion, both CZA and AZA showed good *in vitro* antibacterial activity against BSIs-CROs in China. In addition, CZA showed a higher susceptibility to CR-Kpn and CR-Pae, while AZA was highly active against CRE. Furthermore, the *in vitro* activity of CZA and AZA was improved against CROs with the increase of avibactam concentration. Rational strategies need to be confirmed in further prospective studies.

## Data Availability Statement

The original contributions presented in the study are included in the article/**Supplementary Material**. Further inquiries can be directed to the corresponding author.

## Ethics Statement

In our study, we did not perform any experiments with animals or higher invertebrates, nor performed experiments on humans or the use of human tissue samples.

## Author Contributions

WY and YX developed the concept. WY and LX designed the experiments. JJ, CY, and ZL performed the laboratory measurements. WY and QL analyzed the data. YC and YX gave conceptual advice. WY and YX wrote the paper. All authors discussed the results and implications and commented on the manuscript at all stages. All authors contributed to the article and approved the submitted version.

## Funding

This study was funded by the Key Research and Development Program of Zhejiang Province (no. 2021C03068) and the Youth Program of National Natural Science Foundation of China (no. 81803589). The funder had no role in the study design, data collection and analysis, decision to publish, or preparation of the manuscript.

## Conflict of Interest

The authors declare that the research was conducted in the absence of any commercial or financial relationships that could be construed as a potential conflict of interest.

## Publisher’s Note

All claims expressed in this article are solely those of the authors and do not necessarily represent those of their affiliated organizations, or those of the publisher, the editors and the reviewers. Any product that may be evaluated in this article, or claim that may be made by its manufacturer, is not guaranteed or endorsed by the publisher.

## Supplementary Material

Supplementary Figure 1 Cumulative inhibition ratio (CIR) of CAZ, CZA, ATM and AZA against CROs. **(A)** CAZ and CZA against CR-Eco; **(B)** ATM and AZA against CR-Eco; **(C)** CAZ and CZA against CR-Kpn; **(D)** ATM and AZA against CR-Kpn; **(E)** CAZ and CZA against CR-Pae; **(F)** ATM and AZA against CR-Pae.
