# Supplementary figures and images for: In Vitro Activity Comparison of Ceftazidime–Avibactam and Aztreonam–Avibactam Against Bloodstream Infections With Carbapenem-Resistant Organisms in China
Source: Front Cell Infect Microbiol. 2021 Nov 25;11:780365. doi: 10.3389/fcimb.2021.780365 (PMC8656719; doi:10.3389/fcimb.2021.780365)

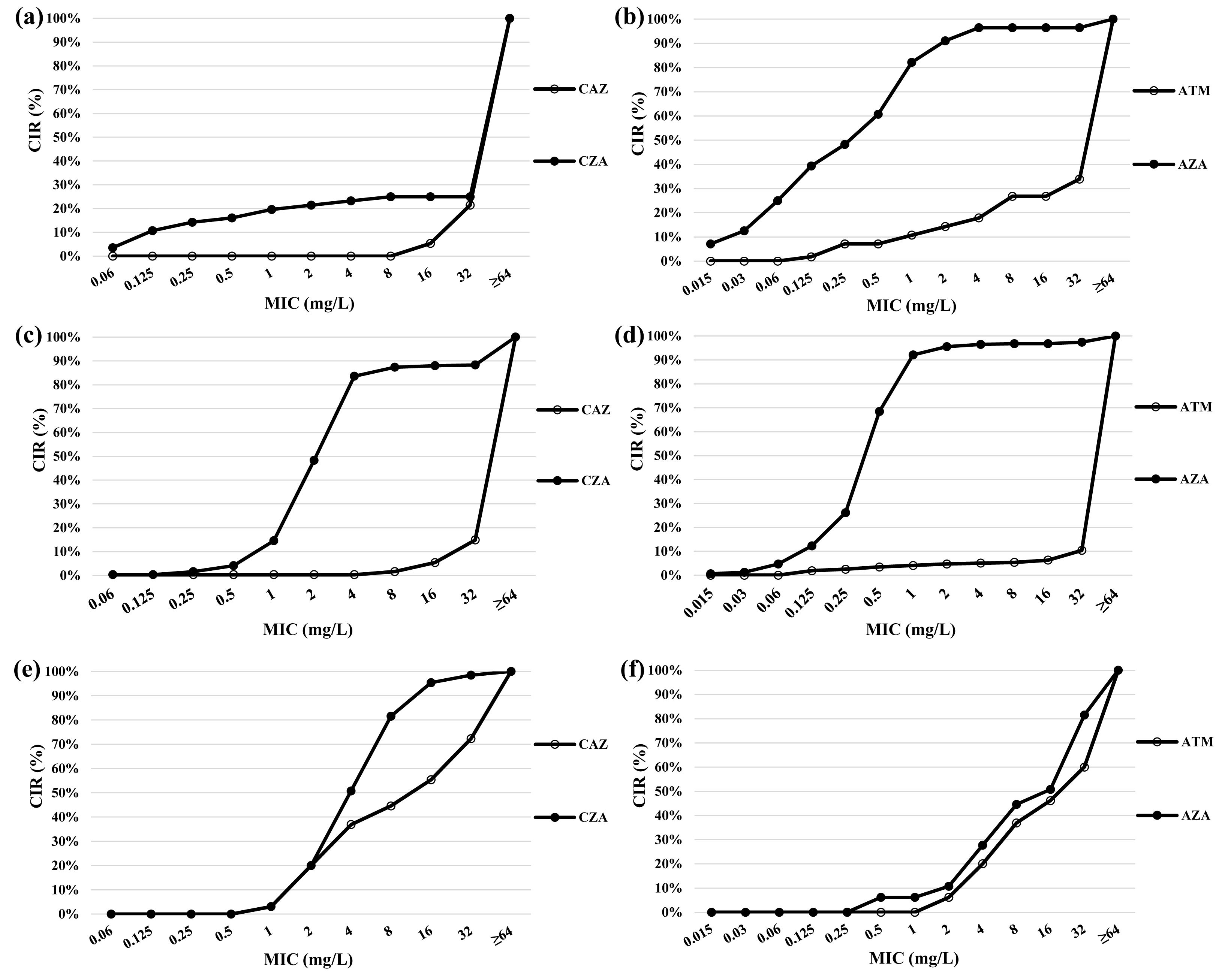

Supplement: Supplementary Figure 1 — Cumulative inhibition ratio (CIR) of CAZ, CZA, ATM and AZA against CROs. (A) CAZ and CZA against CR-Eco; (B) ATM and AZA against CR-Eco; (C) CAZ and CZA against CR-Kpn; (D) ATM and AZA against CR-Kpn; (E) CAZ and CZA against CR-Pae; (F) ATM and AZA against CR-Pae. [file Image_1.tif]
