# Supplementary material for: In Vitro Activity Comparison of Ceftazidime–Avibactam and Aztreonam–Avibactam Against Bloodstream Infections With Carbapenem-Resistant Organisms in China
Source: Front Cell Infect Microbiol. 2021 Nov 25;11:780365. doi: 10.3389/fcimb.2021.780365 (PMC8656719; doi:10.3389/fcimb.2021.780365)
Supplement: Supplementary file 2 [file Table_1.docx]

**Supplementary table 1.** Genotype of CZA-resistant CROs

| Carbapenemase genes | CZA-resistant CROs | | |
| --- | --- | --- | --- |
|  | CR-Eco (N=42) | CR-Kpn (N=40) | CR-Pae (N=12) |
| *bla*_KPC_ | 0 | 14 | 4 |
| *bla*_NDM_ | 39 | 16 | 0 |
| *bla*_IMP_ | 0 | 3 | 1 |
| Two carbapenemase genes | 3 (*bla*_IMP+_*bla*_NDM_) | 2 (*bla*_IMP_+*bla*_NDM_)  3 (*bla*_KPC+_*bla*_NDM_) | 0 |
| No carbapenemase | 0 | 2 | 7 |

CZA, ceftazidime-avibactam; CROs, carbapenem-resistant organisms; CR-Eco, carbapenem-resistant *Escherichia coli*, CR-Kpn; carbapenem-resistant *Klebsiella pneumoniae*; CR-Pae, carbapenem-resistant *Pseudomonas aeruginosa*.
